# Clinical outcome of patients with metastatic melanoma of unknown primary in the era of novel therapy

**DOI:** 10.1007/s00262-021-02871-1

**Published:** 2021-03-27

**Authors:** Danielle Verver, Dirk J. Grünhagen, Alexander C. J. van Akkooi, Maureen J. B. Aarts, Franchette W. P. J. van den Berkmortel, Alfonsus J. M. van den Eertwegh, Jan Willem B. de Groot, Marye J. Boers-Sonderen, John B. A. G. Haanen, Geke A. P. Hospers, Ellen Kapiteijn, Djura Piersma, Rozemarijn S. van Rijn, Karijn P. M. Suijkerbuijk, Albert J.ten Tije, Gerard Vreugdenhil, Cornelis Verhoef, Astrid A. M. van der Veldt

**Affiliations:** 1 Department of Surgical Oncology, Erasmus MC Cancer Institute, Dr Molewaterplein 40, 3015 GD Rotterdam, The Netherlands; 2 Department of Surgical Oncology, Netherlands Cancer Institute–Antoni van Leeuwenhoek, Amsterdam, The Netherlands; 3 Department of Medical Oncology, Maastricht University Medical Centre+, Maastricht, The Netherlands; 4 Department of Medical Oncology, Zuyderland Medical Centre, Sittard-Geleen, The Netherlands; 5 Department of Medical Oncology, Amsterdam UMC, Location VU University Medical Centre (VUmc), Cancer Centre Amsterdam, Amsterdam, The Netherlands; 6 Oncology Centre Isala, Isala, Zwolle, The Netherlands; 7 Department of Medical Oncology, Radboud University Medical Centre, Nijmegen, The Netherlands; 8 Department of Medical Oncology, Netherlands Cancer Institute, Amsterdam, The Netherlands; 9 Department of Medical Oncology, University Medical Centre Groningen, University of Groningen, Groningen, The Netherlands; 10 Department of Medical Oncology, Leiden University Medical Centre, Leiden, The Netherlands; 11 Department of Internal Medicine, Medisch Spectrum Twente, Enschede, The Netherlands; 12 Department of Internal Medicine, Medical Centre Leeuwarden, Leeuwarden, The Netherlands; 13 Department of Medical Oncology, University Medical Centre Utrecht Cancer Centre, Utrecht, The Netherlands; 14 Department of Internal Medicine, Amphia Hospital, Breda, The Netherlands; 15 Department of Internal Medicine, Maxima Medical Centre, Eindhoven, The Netherlands; 16 Departments of Medical Oncology and Radiology and Nuclear Medicine, Erasmus MC Cancer Institute, Rotterdam, The Netherlands

**Keywords:** Melanoma, Unknown primary, Known primary, Novel therapy

## Abstract

Melanoma of unknown primary (MUP) is considered different from melanoma of known primary (MKP), and it is unclear whether these patients benefit equally from novel therapies. In the current study, characteristics and overall survival (OS) of patients with advanced and metastatic MUP and MKP were compared in the era of novel therapy. Patients were selected from the prospective nation-wide Dutch Melanoma Treatment Registry (DMTR). The following criteria were applied: diagnosis of stage IIIc unresectable or IV cutaneous MKP (cMKP) or MUP between July 2012 and July 2017 and treatment with immune checkpoint inhibition and/or targeted therapy. OS was estimated using the Kaplan–Meier method. The stratified multivariable Cox regression model was used for adjusted analysis. A total of 2706 patients were eligible including 2321 (85.8%) patients with cMKP and 385 (14.2%) with MUP. In comparative analysis, MUP patients more often presented with advanced and metastatic disease at primary diagnosis with poorer performance status, higher LDH, and central nervous system metastases. In crude analysis, median OS of cMKP or MUP patients was 12 months (interquartile range [IQR] 5 – 44) and 14 months (IQR 5 – not reached), respectively (*P* = 0.278). In adjusted analysis, OS in MUP patients was superior (hazard rate 0.70, 95% confidence interval 0.58–0.85; *P* < 0.001). As compared to patients with advanced and metastatic cMKP, MUP patients have superior survival in adjusted analysis, but usually present with poorer prognostic characteristics. In crude analysis, OS was comparable indicating that patients with MUP benefit at least equally from treatment with novel therapies.

## Introduction

Melanoma of unknown primary (MUP) is rare, as only 3% of all melanoma patients present with stage I-IV MUP [1]. Patients with MUP usually present with (presumed) locoregional melanoma metastases in the (sub)cutis, soft tissue, and/or lymph nodes (i.e. stage III disease) or with distant metastases including visceral metastases (i.e. stage IV disease) [2].

To date, the origin of MUP has still not been unravelled. Possible explanations include unrecognised melanomas, (traumatically) removed melanomas without pathological review, the development of de novo melanomas within lymph nodes and/or at other non-cutaneous sites, and missed diagnosis of spontaneous regressing melanomas [3,4]. This latter explanation is supported by several studies observing regressed pigmented lesions in patients with MUP [5–9]. Spontaneous regression, especially partial spontaneous regression, is rather common in melanoma [[10]. As an enhanced immune response with an increased number of tumour infiltrating T lymphocytes can be found in regressing melanoma, spontaneous regression is considered the result of an effective host immune response [11,12]. Although the prognostic significance of melanoma regression remains controversial [13], it seems to be associated with favourable prognosis[12]. As MUP may originate from primary melanomas with immune-mediated spontaneous regression, MUP may have a different biology with immunological surveillance mechanisms. As a result, patients with MUP may have a more favourable prognosis as compared to patients with melanoma of known primary (MKP). This hypothesis is supported by a meta-analysis which was conducted before the introduction of novel therapies. In this meta-analysis, patients with stage IV MUP had improved overall survival (OS) as compared with patients with stage IV MKP [14].

Survival of patients with advanced and metastatic MKP and MUP has significantly improved since the introduction of novel therapies, including immune checkpoint inhibition (ICI) and targeted therapy [1,15]. Immune checkpoint inhibitors are monoclonal antibodies that enhance anti-tumour T-cell-mediated immune responses by releasing their suppression by immune-checkpoints like cytotoxic T-lymphocyte-associated protein 4 (CTLA-4; e.g. ipilimumab) [16,17] or programmed death-1 (PD-1) receptor (e.g. nivolumab, pembrolizumab) [18–21]. For the treatment of advanced and metastatic melanoma, monotherapy anti-PD1 and combination therapy with ipilimumab and nivolumab has also been approved[22]. Targeted therapy has a different mechanism of action and blocks cancer cell proliferation by selective BRAF inhibitors (BRAFi, i.e. vemurafenib, dabrafenib, encorafenib) and MEK inhibitors (MEKi, i.e. trametinib, cobimetinib, binimetinib) [23–29]. Approximately half of the patients with cutaneous melanoma have benefit from these targeted therapies, which is determined by the presence of a tumour mutation at codon V600 of the BRAF gene. However, targeted therapies may have a more extensive mechanism of action, as these agents are also known to induce immune responses [30].

Until now, information on survival outcomes in patients with MUP treated with these novel therapies is lacking, as clinical trials have not reported on patients with MUP specifically, although they might have been included. Based on the immunological surveillance hypothesis, patients with MUP may derive more benefit from these novel therapies, in particular, since both ICI and targeted therapy have the potential to enhance the anti-tumour immune response [30]. In the current study, patients’ characteristics and OS were compared between MUP and MKP patients treated with novel therapies within a large nation-wide prospective Dutch cohort.

## Methods

### Data

Data were retrieved from the Dutch Melanoma Treatment Registry (DMTR), a population-based registry that was initiated in July 2013 to assess the quality of melanoma care in the Netherlands. In the DMTR, safety and efficacy of novel therapies are monitored in real-world clinical practice. Prospective registration started from July 2013. Between July 2012 and July 2013, data were collected retrospectively. The DMTR documents detailed information on all Dutch patients with stage IIIc^unresectable^ or IV melanoma (advanced and metastatic melanoma), including tumour and patient characteristics, treatment patterns, and clinical outcomes. A detailed description of the DMTR has been published previously [31].

### Patients

For inclusion in the analysis, patients had to fulfil the following inclusion criteria: age > 18 years, MUP or cutaneous MKP (cMKP), diagnosis of stage IIIc unresectable or IV melanoma between July 2012 and July 2017, and treatment with novel systemic therapy (i.e. ICI and/or targeted therapy) during any of the registered treatment episodes. Melanoma with regional and/or distant metastasis without a primary melanoma was categorised as MUP. Novel systemic therapy included: BRAFi, BRAFi plus MEKi, anti-CTLA-4 monotherapy (ipilimumab), anti-PD1 monotherapy (nivolumab or pembrolizumab), and combination therapy of ipilimumab and nivolumab. Data on pre-novel therapy (i.e. other treatment after diagnosis of stage IIIc unresectable or IV melanoma and prior to initiation of novel systemic therapy) were also collected and included local therapy (i.e. surgery and radiotherapy) or other systemic therapy (e.g. chemotherapy). Treatment other than novel systemic therapy prior to diagnosis of stage IIIc unresectable or IV disease, or after initiation of novel systemic therapy were not included in the analyses. Cut-off of follow-up data was set at April 1st 2018.

According to time interval, melanoma was categorised into primary advanced and metastatic disease (i.e. diagnosis of stage IIIc unresectable or IV melanoma ≤ 3 months after first pathological melanoma diagnosis) and secondary advanced and metastatic disease (i.e. diagnosis of stage IIIc unresectable or IV melanoma > 3 months after first pathological diagnosis of melanoma). In addition, melanoma specific mutations were categorised into BRAF V600E/K mutation present, absent or unknown. Other BRAF mutations (i.e. non-BRAF V600E/K) were categorised as absent. The following patients’ and disease characteristics were collected at initiation of first-line novel therapy: age, Eastern Cooperative Oncology Group (ECOG) performance status, number of metastatic sites, central nervous system (CNS) metastases, and serum lactate dehydrogenase (LDH).

As most benefit was expected from ICI, subgroup analyses were performed for patients ever treated with anti-PD1 therapy (including monotherapy and combination with anti-CTLA). In addition, survival analyses were performed for patients treated with BRAF inhibitors (BRAFi), BRAFi plus MEK inhibitors (MEKi), ipilimumab monotherapy, anti-PD1 monotherapy, or combination therapy with ipilimumab and nivolumab. Treatment strategy was categorised as first-line therapy (‘first’), only line (‘only’), and at any time (‘ever’).

### Outcomes

The primary outcome measure was OS. The OS time was defined from start date of first-line novel therapy to last date of follow-up or death by any cause.

### Statistical analysis

Data were presented as prevalence (percentage) or median (interquartile range [IQR]). Differences between groups were calculated using chi-square tests, Fisher exact tests or non-parametric Mann–Whitney *U* tests. Among survivors, the median duration of follow-up was calculated from date of initiation of novel therapy to date of last follow-up using the reversed Kaplan–Meier method (deaths were censored). Crude (unadjusted) OS was estimated using the Kaplan–Meier method and presented in median with IQR. The log rank test was used to compare survival. Only available data were analysed with listwise deletion in multivariable analysis. For adjusted analysis, a multivariable Cox proportional hazards regression analysis was used to assess the effect of several potential prognostic factors on OS. Based on literature review and availability of sufficient data, the following variables were identified as potential prognostic factors: gender, origin of melanoma (cMKP or MUP), timing of metastasis (i.e. primary versus secondary advanced and metastatic disease), BRAF V600E/K mutation status in melanoma, pre-novel therapy (i.e. treatment other than novel systemic therapy initiated after diagnosis of advanced and metastatic disease and prior to initiation of novel systemic therapy), clinical characteristics at start of novel therapy (age, ECOG performance status, serum LDH level, CNS metastases), and treatment with anti-PD1 therapy (i.e. monotherapy and combination) [2,3,32–36]. The proportional hazards assumption was tested by correlating the corresponding set of scaled Schoenfeld residuals with time, thereby testing for independence between residuals and time. For variables affecting the proportional hazards assumption, the stratified Cox procedure was used. *P* values ≤ 0.05 were considered statistically significant. All statistical analyses were performed using SPSS version 25.0 (IBM, Armonk, NEW York, USA) and R (version 3.6.1, R Foundation for Statistical Computing, Vienna, Austria, 2019).

## Results

### Patient characteristics

**Fig. 1:**
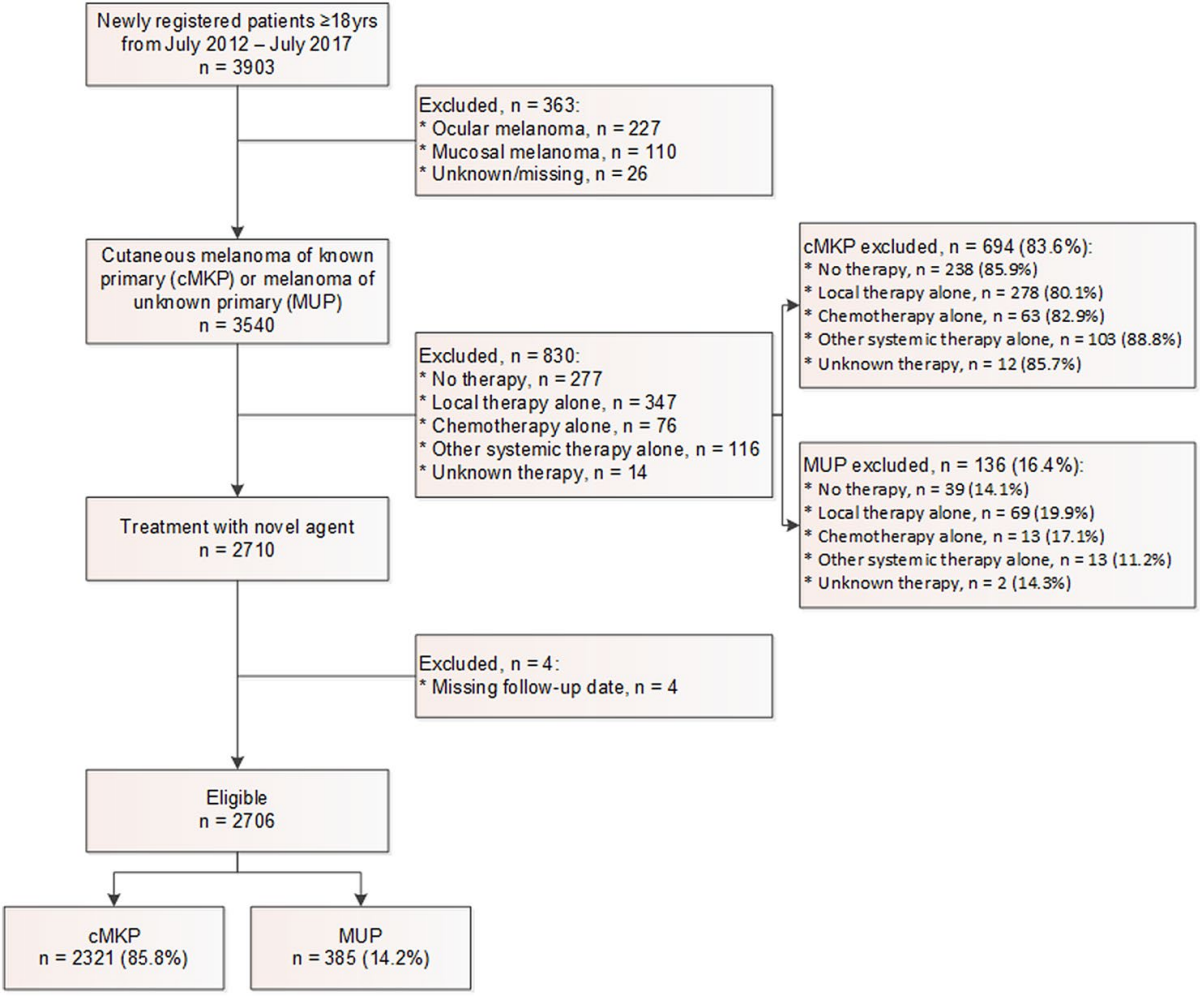
Flow diagram of patient selection

Between July 2012 and July 2017, 3903 patients (age ≥ 18 years) with advanced and metastatic melanoma were registered in the Netherlands. After exclusion, a total of 2706 out of 3903 patients were eligible for the current study including 2321 patients with cMKP (85.8%) and 385 patients with MUP (14.2%) (Fig. 1). For all survivors, the median follow-up was 24 months (IQR 14 – 35).

At primary diagnosis, patients with MUP more often presented with advanced and metastatic disease (i.e. stage IIIc^unresectable^ or IV melanoma) as compared with patients with cMKP (72.5% versus 7.3%, respectively, *P* < 0.001). In addition, patients with MUP more frequently presented with significantly worse ECOG performance status and CNS metastases (Table 1). BRAF V600E/K mutation was present in 59.2% and 54.3% of patients with cMKP and MUP, respectively (*P* = 0.038). Among patients with cMKP and MUP, pre-novel therapy was significantly different (*P* = 0.043), as more patients with cMKP received systemic therapy (e.g. chemotherapy; 5.6% versus 2.9%) (Table 1).

**Table 1:** Comparative analysis of patient, disease and treatment characteristics

All patients				Anti-PD1 therapy ever (monotherapy and combination)		
Characteristics	cMKP (*n* = 2321)	MUP (*n* = 385)	*P*	cMKP (*n* = 1150)	MUP (*n* = 196)	*P*
Age, yrs	62 (52—71)	61 (53—69)	0.108	63 (53—71)	62 (53—69)	0.459
Gender			0.368^$^			0.378^$^
Male	1362 (58.7)	238 (61.8)		671 (58.3)	123 (62.8)	
Female	958 (41.3)	147 (38.2)		478 (41.6)	73 (37.2)	
Unknown	1 (0.1)	0		1 (0.1)	0	
Timing advanced and metastatic disease			< 0.001			< 0.001
Primary	169 (7.3)	279 (72.5)		84 (7.3)	154 (78.6)	
Secondary	2152 (92.7)	106 (27.5)		1066 (92.7)	42 (21.4)	
ECOG performance			0.004			0.044
0	1191 (51.3)	163 (42.3)		680 (64.0)	99 (54.4)	
≥ 1	929 (40.0)	186 (48.3)		382 (36.0)	83 (45.6)	
Unknown	201 (8.7)	36 (9.4)		88 (7.7)	14 (7.1)	
LDH value			0.096			0.013
Normal	1428 (61.5)	220 (57.1)		767 (66.7)	110 (56.1)	
Elevated	836 (36.0)	159 (41.3)		358 (31.1)	82 (41.8)	
Unknown	57 (2.5)	6 (1.6)		25 (2.2)	4 (2.0)	
CNS metastases			0.001			0.002
Absent	1552 (66.9)	250 (64.9)		807 (70.2)	133 (67.9)	
Present	595 (25.6)	123 (31.9)		254 (22.1)	59 (30.1)	
Unknown	174 (7.5)	12 (3.1)		89 (7.7)	4 (2.0)	
No. of metastases independent of location			0.836			0.582
< 5	292 (12.6)	52 (13.5)		171 (14.9)	27 (13.8)	
5 – 10	141 (6.1)	26 (6.8)		86 (7.5)	11 (5.6)	
> 10	1576 (67.9)	260 (67.5)		701 (61.0)	129 (65.8)	
Unknown	312 (13.4)	47 (12.2)		192 (16.7)	29 (14.8)	
BRAF V600E/K mutation			0.038			0.114
Absent	848 (36.5)	150 (39.0)		536 (46.6)	100 (51.0)	
Present	1375 (59.2)	209 (54.3)		565 (49.1)	83 (42.3)	
Unknown	98 (4.2)	26 (6.8)		49 (4.3)	13 (6.6)	
Pre-novel therapy^@^			0.043			0.232
None	2020 (87.0)	339 (88.1)		993 (86.3)	167 (85.2)	
Local therapy (e.g. surgery, radiotherapy)	170 (7.3)	35 (9.1)		113 (9.8)	25 (12.8)	
Systemic therapy (e.g. chemotherapy, other)	131 (5.6)	11 (2.9)		44 (3.8)	4 (2.0)	
Novel therapy first-line			0.819			0.966
First-line BRAFi	711 (30.6)	106 (27.5)		113 (9.8)	22 (11.2)	
First-line BRAFi + MEKi	367 (15.8)	65 (16.9)		150 (13.0)	25 (12.8)	
First-line ipi	574 (24.7)	99 (25.7)		218 (19.0)	34 (17.3)	
First-line anti-PD1 mono	593 (25.5)	102 (26.5)		593 (51.6)	102 (52.0)	
First-line ipi + nivo	76 (3.3)	13 (3.4)		76 (6.6)	13 (6.6)	
Novel therapy only			0.691			0.897
BRAFi only	417 (18.0)	58 (15.1)		n/a	n/a	
BRAFi + MEKi only	168 (7.2)	32 (8.3)		n/a	n/a	
Ipi only	291 (12.5)	54 (14.0)		n/a	n/a	
Anti-PD1 mono only	422 (18.2)	75 (19.5)		422 (36.7)	75 (38.3)	
Ipi + nivo only	58 (2.5)	9 (2.3)		58 (5.0)	9 (4.6)	
Novel therapy combinations^	965 (41.6)	157 (40.8)		670 (58.3)	112 (57.1)	
Novel therapy ever^#^						
BRAFi ever	824 (35.5)	121 (31.4)	0.120	167 (14.5)	30 (15.3)	0.774
BRAFi + MEKi ever	586 (25.2)	102 (26.5)	0.603	328 (28.5)	55 (28.1)	0.895
Ipi ever	849 (36.6)	147 (38.2)	0.546	346 (30.1)	60 (30.6)	0.882
Anti-PD1 mono ever	1022 (44.0)	178 (46.2)	0.421	1022 (88.9)	196 (90.8)	0.418
Ipi + nivo ever	156 (6.7)	23 (6.0)	0.585	156 (13.6)	23 (11.7)	0.485
No. of novel therapy lines			0.357			0.295
One line	1356 (58.4)	228 (59.2)		480 (41.7)	84 (42.9)	
Two line	642 (27.7)	93 (24.2)		416 (36.2)	59 (30.1)	
Three lines	215 (9.3)	43 (11.2)		159 (13.8)	32 (16.3)	
> Three lines	108 (4.7)	21 (5.5)		95 (8.3)	21 (10.7)	

*BRAF*i BRAF inhibition; *CNS *central nervous system; *Ipi* ipilimumab; *LDH* lactate dehydrogenase; *MEKi* MEK inhibition; *Nivo* nivolumab;

$Fisher exact test

^@^After diagnosis of advanced and metastatic disease but prior to initiation of novel therapy

#percentage yes per category

^Concerns patients who received more than one line of novel therapy

### Novel therapy

Time from diagnosis of stage IIIc unresectable and IV melanoma to initiation of first-line novel therapy was not different between MUP and cMKP patients (1 month (IQR 0 -2) and 1 month (IQR 0 – 2), respectively, *P* = 0.444). Applied novel therapy strategies (first, only, and ever) for both ICI and targeted therapy, as well as the total number of novel therapy lines were similar for patients with MUP and cMKP (Table 1). Overall, 1150 patients with cMKP (49.5%) and 196 patients with MUP (50.9%) ever received anti-PD1 therapy for stage IIIc unresectable or IV melanoma. In this subgroup, BRAF V600E/K mutation was comparable in cMKP and MUP patients (46.6% vs. 51.0%, P = 0.114), whereas patients with MUP more frequently presented with advanced and metastatic disease at primary diagnosis with worse ECOG performance status, higher LDH, and CNS metastases (Table 1).

### Survival

**Fig. 2:**
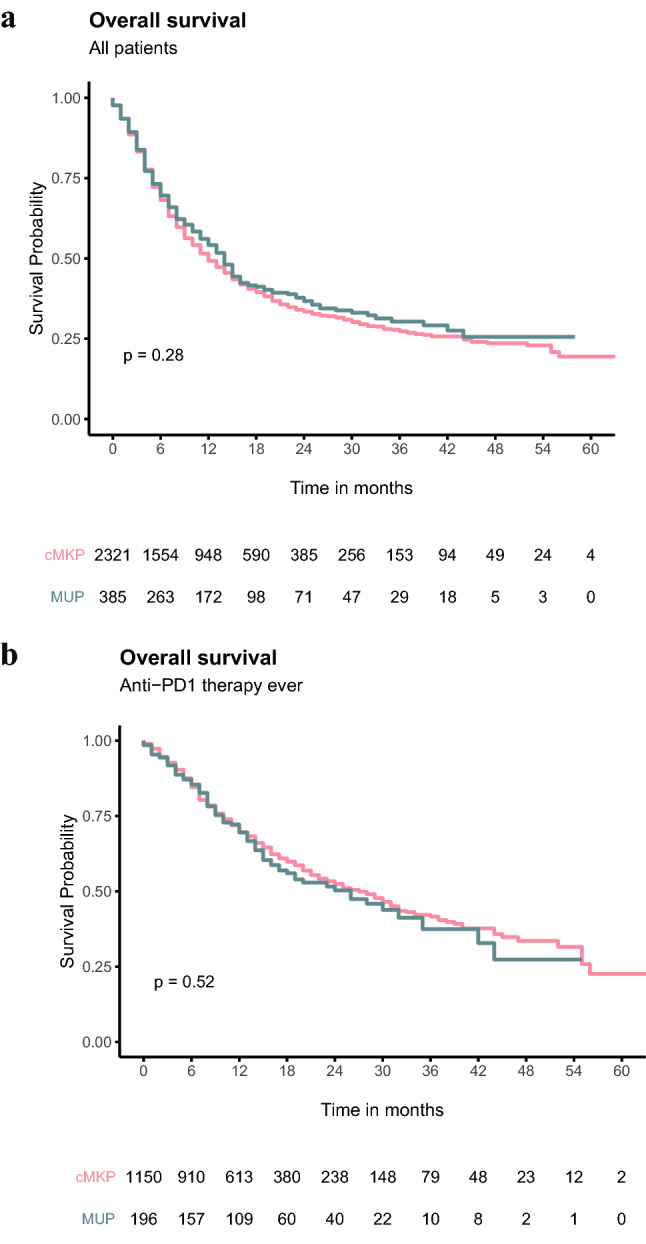
Crude survival in **a** all patients and **b** patients ever treated with anti-PD1 therapy (monotherapy and combination)

**Fig. 3:**
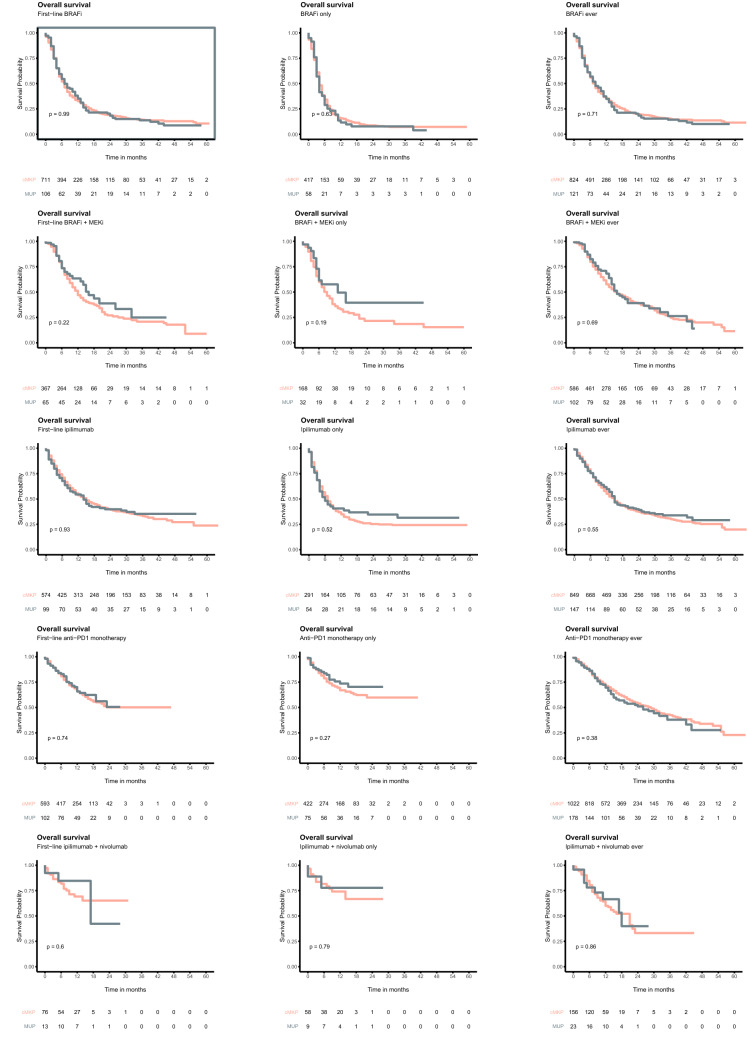
Crude OS in patients with MKP and MUP treated first-line, only or ever with BRAFi monotherapy, BRAFi plus MEKi, ipilimumab monotherapy, anti-PD1 monotherapy, and combination of ipilimumab and nivolumab

In crude analysis, patients with cMKP and MUP had comparable median OS of 12 months (IQR, 5 – 44) and 14 months (IQR, 5 – not reached), respectively (*P* = 0.28; Fig. 2a). In the subgroup of patients ever treated with anti-PD1 therapy for stage IIIc unresectable or IV, a comparable median OS of 27 months (IQR, 10 – 56) and 26 months, (IQR 10 – not reached) was measured in patients with cMKP and MUP (P = 0.52), respectively (Fig. 2b). In addition, OS was not different for all other strategies of administered novel therapy (i.e. BRAFi, BRAFi plus MEKi, ipilimumab monotherapy, anti-PD1 monotherapy or ipilimumab + nivolumab) as first, only, and ever treatment line (Fig. 3.).

**Fig. 4:**
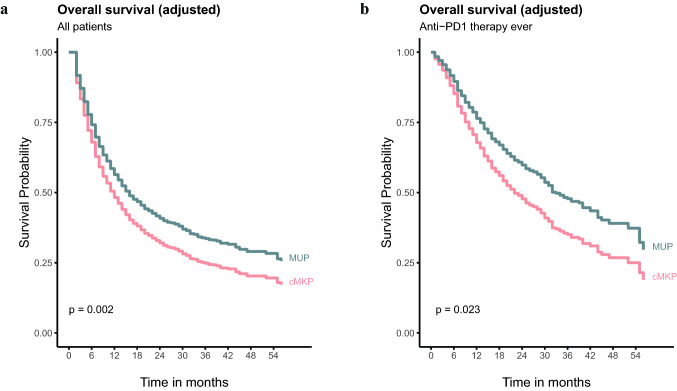
Adjusted (expected) survival from start of novel therapy based on the multivariable cox models in **a** all patients and **b** patients ever treated with anti-PD1 therapy (monotherapy and combined)

In multivariable analysis, patients with MUP had improved OS as compared to patients with cMKP, when adjusted for age, gender, CNS metastases, timing of metastasis, pre-novel therapy, and stratified (due to affecting the proportional hazards assumption) for ECOG performance, LDH, BRAF V600E/K mutation, and anti-PD1 therapy (hazard rate 0.74, 95% confidence interval (CI) 0.61 – 0.90; *P* = 0.002) (Table 2). In patients ever treated with anti-PD1 therapy, OS was improved in patients with MUP as compared to patients with cMKP, when adjusted for age, gender, timing of metastasis, ECOG performance status, CNS metastases, pre-novel therapy, ipilimumab combined with nivolumab therapy, and stratified for serum LDH and BRAF V600E/K mutation (hazard rate 0.87, 95% CI 0.48 – 0.96; *P* = 0.028) (Table 2). The adjusted expected survival curves of the analyses are shown in Fig. 4.

**Table 2:** Stratified Cox regression models for overall survival for all patients and according to anti-PD1 therapy ever (monotherapy and combination)

All patients^a^			Anti-PD1 therapy^b^	
	HR (95% CI)	*P* value	HR (95% CI)	*P* value
Origin				
cMKP	Reference		Reference	
MUP	0.74 (0.61—0.90)	0.002	0.87 (0.48—0.96)	0.028
Age, yrs	1.01 (1.00—1.01)	0.017	1.01 (1.01—1.02)	0.001
*Gender*				
Male	Reference		Reference	
Female	0.86 (0.77—0.96)	0.010	0.97 (0.80—1.18)	0.766
*Timing advanced and metastatic disease*				
Primary	Reference		Reference	
Secondary	0.84 (0.70—1.01)	0.066	0.67 (0.49—0.91)	0.010
*ECOG performance*				
0	n/a	n/a	Reference	
> 0	n/a	n/a	1.47 (1.21—1.78)	< 0.001
*CNS metastases*				
No	Reference		Reference	
Yes	1.65 (1.46—1.86)	< 0.001	1.71 (1.39—2.10)	< 0.001
*Pre-novel therapy*^*c*^				
None	Reference		Reference	
Local therapy	0.88 (0.68—1.14)	0.325	0.78 (0.54—1.13)	0.189
Systemic therapy	0.94 (0.73—1.20)	0.618	1.11 (0.69—1.78)	0.680
*Ipi + nivo ever*				
No	n/a	n/a	Reference	
Yes	n/a	n/a	1.35 (1.01—1.79)	0.040

*CNS* central nervous system; *n/a* not applicable

^a^Stratified by serum level LDH, BRAF V600E/K, ECOG performance and anti-PD1 therapy status

^b^Stratified by serum level LDH and BRAF V600E/K

^c^After diagnosis of advanced and metastatic disease but prior to initiation of novel therapy

### Discussion

To date, this study represents the largest study in patients with advanced and metastatic MUP and cMKP in the novel therapy era. For stage IIIc unresectable and IV, survival advantage was measured for patients with MUP as compared to patients with cMKP, when adjusted for several prognostic factors. However, in crude analysis, OS was similar, even in patients ever treated with anti-PD1 therapy.

MUP is considered relatively rare as approximately 3% of all patients with newly diagnosed melanoma stage I-IV present with MUP [1]. However, MUP is more common in patients with advanced and metastatic melanoma. In the current study, approximately 14% of all patients with stage IIIc^unresectable^ and IV melanoma were diagnosed with MUP. These results are supported by similar rates in other reports [37]. We previously demonstrated that the introduction of novel therapies for patients with (primary) advanced and metastatic MUP has led to a significantly improved median OS from 4 to 11 months [1]. The current study focusses on the relevant question whether this survival benefit is similar for patients with MUP as compared to patients with cMKP. To this end, a large nation-wide prospective Dutch cohort was analysed, thereby including both patients with primary and secondary advanced and metastatic disease.

Compared to patients with cMKP, patients with MUP more frequently presented with poorer prognostic factors, including advanced and metastatic melanoma at primary diagnosis, higher ECOG performance status, higher LDH, and CNS metastases. Interestingly, despite these poorer prognostic factors, patients with MUP had comparable OS in crude analysis. This may suggest that patients with MUP have favourable factors which are still unknown. In adjusted analysis, correcting for the known poorer prognostic factors, patients with MUP show improved OS. In another large study, conducted before the introduction of novel therapies, patients with MUP also had similar OS in crude analysis and improved survival in adjusted analysis [38]. These findings suggest that the possible favourable factors in patients with MUP are not affected by novel therapy. Based on the immunological surveillance hypothesis, a larger benefit from novel therapies, especially ICI, may have been expected in patients with MUP thus resulting in improved survival even in the unadjusted analysis.

Our results are supported by a recent Danish study in 576 patients comparing survival between patients with cMKP (*n* = 496) and MUP (*n* = 80) after the introduction of novel therapies [39]. In this Danish analysis, approximately 40% of the included patients had relatively good prognostic factors including ECOG 0 – 1, normal LDH, and absence of active CNS metastases. Patients with MUP showed poorer prognostic factors in terms of disease stage. Nevertheless, OS was comparable in crude analysis, with median OS of 9.7 and 10.0 months for patients with cMKP and MUP (*P* = 0.84), respectively. The imbalance in disease stage partly explains the non-superior survival for patients with MUP [40]. The observed lower median survival may be explained by the fact that patients who were not treated with novel therapies (or not treated at all) were also included. Another recent small pilot study showed different results in 41 patients treated with ICI [41]. The patient population was small and included a relatively high number of patients with MUP (22%) with comparable baseline characteristics as patients with MKP. Although this population may not be representative of real-world MUP patients, the pilot study showed an OS benefit in MUP patients treated with ICI.

In clinical trials on novel therapy, outcomes for patients with MUP have not been reported, and it is unclear how many patients with MUP were included. Although MUP was not an exclusion criterion, it is likely that most patients with MUP were ineligible based on other criteria such as ECOG performance status > 1, elevated LDH, and presence of (symptomatic) CNS metastases. As demonstrated in the current study, patients with MUP at least have similar benefit from treatment with novel agents, despite these poorer prognostic factors.

Overall, patients with cMKP and MUP were treated according to similar strategies. Also, for the excluded patients who did not receive novel therapy the distribution of cMKP and MUP was largely similar (Fig. 1). Noteworthy, approximately half of the patients in both groups ever received anti-PD1 therapy during the course of treatment. This relatively limited use of anti-PD1 therapy is presumably related to the years of approval, availability and/or incorporation in Dutch guidelines. In addition, some patients might have had long-term benefit from targeted therapy without the need for anti-PD1 therapy, whereas other patients might have had rapid progressive disease while anti-PD1 monotherapy or the combination was not available.

The current study has several limitations of which some are inherently related to the registration of real-world data such as incomplete data. Therefore, it is conceivable that some cases of MUP may have been misclassified, as it was not registered whether patients with MUP had exclusion criteria for diagnosis of MUP, including prior orbital exenteration or enucleation, prior skin excision, electrodessication, cauterization, or other surgical manipulation of a mole, freckle, birthmark, paronychia, or skin blemish [42]. In addition, some MUPs may have been misclassified because of limited diagnostics, since the Dutch Melanoma guidelines do not recommend endoscopy, ophthalmoscopy and/or nasopharyngoscopy. Unfortunately, information on these examinations was not available in the DMTR. On the other hand, extensive diagnostic imaging is usually performed in patients with MUP, as these patients primarily present with stage IIIb-IV. In the current study, data on positron emission tomography (PET) were only available at the time of initial staging of IIIc unresectable and IV melanoma. As 72.5% of MUP patients primarily presented with stage IIIcunresectable and IV melanoma and 65.6% of these patients underwent PET at initial staging, misclassification of MUP is probably limited. Another limitation may be the presence of lead-time bias due to different disease patterns of MUP and cMKP, with advanced and metastatic disease already present at time of primary diagnosis of MUP in most cases. Therefore, it is conceivable that advanced and metastatic disease was detected earlier in patients with cMKP as a result of patient awareness and active surveillance after primary diagnosis of cMKP. Another potential limitation is the limited follow-up period for newer agents, potentially resulting in less representative survival data for patients treated with these agents. Finally, patients with non-cutaneous MKP (i.e. ocular and mucosal primary melanoma) were excluded in order to generate a more homogenous population, although it is unknown whether MUP may have its origin in non-cutaneous sites. On the other hand, the genotypes of MUP and cMKP are comparable, indicating that MUP most likely arises from (regressed) cutaneous sites [43–45].

In conclusion, as compared to patients with advanced and metastatic cMKP, patients with MUP have comparable overall survival in crude analysis and show superior survival in adjusted analysis. This indicates that patients with MUP benefit at least equally from treatment with novel therapies, although they usually present with poorer prognostic factors. Therefore, novel therapy should not be withheld in patients with advanced stage MUP.

## Data Availability

The data that support the findings of this study are available from the Dutch Melanoma Treatment Registry. Restrictions apply to the availability of these data, which were used under licence for this study. Data are available from the corresponding author upon reasonable request with the permission of the Dutch Melanoma Treatment Registry.

## Abbreviations

CNS: Central nervous system
cMKP: Cutaneous melanoma known primary
CTLA-4: Cytotoxic T-lymphocyte-associated protein 4
DMTR: Dutch Melanoma Treatment Registry
ECOG: Eastern Cooperative Oncology Group
ICI: Immune checkpoint inhibition
IQR: Interquartile range
LDH: Lactate dehydrogenase
MKP: Melanoma known primary
MUP: Melanoma unknown primary
OS: Overall survival
PD-1: Programmed death-1
